# High rifampicin peak plasma concentrations accelerate the slow phase of bacterial decline in tuberculosis patients: Evidence for heteroresistance

**DOI:** 10.1371/journal.pcbi.1011000

**Published:** 2023-04-13

**Authors:** Antal Martinecz, Martin J. Boeree, Andreas H. Diacon, Rodney Dawson, Colin Hemez, Rob E. Aarnoutse, Pia Abel zur Wiesch

**Affiliations:** 1 Department of Pharmacy, Faculty of Health Sciences, University of Tromsø, Tromsø, Norway; 2 Center for Infectious Disease Dynamics, Huck Institutes of the Life Sciences, The Pennsylvania State University, University Park, Pennsylvania, United States of America; 3 Department of Lung Diseases, Radboud Institute for Health Sciences, Radboud university medical center, Nijmegen, the Netherlands; 4 Faculty of Medicine and Health Sciences, Stellenbosch University, Tygerberg, South Africa; 5 TASK Applied Science, Cape Town, South Africa; 6 Division of Pulmonology and Department of Medicine, University of Cape Town, Cape Town, South Africa; 7 University of Cape Town Lung Institute, Cape Town, South Africa; 8 Broad Institute of MIT and Harvard, Cambridge, Massachusetts, United States of America; 9 Graduate program in Biophysics, Harvard University, Boston, Massachusetts, United States of America; 10 Department of Pharmacy, Radboud Institute for Health Sciences, Radboud university medical center, Nijmegen, The Netherlands; 11 Department of Biology, Eberly College of Science, The Pennsylvania State University, University Park, Pennsylvania, United States of America; 12 Norwegian Institute of Public Health (Folkehelseinstitutt), Oslo, Norway; University at Buffalo - The State University of New York, UNITED STATES

## Abstract

**Background:**

Antibiotic treatments are often associated with a late slowdown in bacterial killing. This separates the killing of bacteria into at least two distinct phases: a quick phase followed by a slower phase, the latter of which is linked to treatment success. Current mechanistic explanations for the *in vitro* slowdown are either antibiotic persistence or heteroresistance. Persistence is defined as the switching back and forth between susceptible and non-susceptible states, while heteroresistance is defined as the coexistence of bacteria with heterogeneous susceptibilities. Both are also thought to cause a slowdown in the decline of bacterial populations in patients and therefore complicate and prolong antibiotic treatments. Reduced bacterial death rates over time are also observed within tuberculosis patients, yet the mechanistic reasons for this are unknown and therefore the strategies to mitigate them are also unknown.

**Methods and findings:**

We analyse a dose ranging trial for rifampicin in tuberculosis patients and show that there is a slowdown in the decline of bacteria. We show that the late phase of bacterial killing depends more on the peak drug concentrations than the total drug exposure. We compare these to pharmacokinetic-pharmacodynamic models of rifampicin heteroresistance and persistence. We find that the observation on the slow phase’s dependence on pharmacokinetic measures, specifically peak concentrations are only compatible with models of heteroresistance and incompatible with models of persistence. The quantitative agreement between heteroresistance models and observations is very good ($R_{adj}^{2}=0.97$).

To corroborate the importance of the slowdown, we validate our results by estimating the time to sputum culture conversion and compare the results to a different dose ranging trial.

**Conclusions:**

Our findings indicate that higher doses, specifically higher peak concentrations may be used to optimize rifampicin treatments by accelerating bacterial killing in the slow phase. It adds to the growing body of literature supporting higher rifampicin doses for shortening tuberculosis treatments.

## Author summary

During antibiotic therapy, the rate with which bacteria are eliminated often slows down substantially. This necessitates long treatment duration and increases the risk of relapse after a seemingly successful treatment. The causes of the slowdown can vary depending on the combination of antibiotic and pathogen, and as a result strategies to mitigate this require targeted approaches.

Here, we focus on pulmonary tuberculosis (TB) as it is one of the deadliest diseases with a high burden across the globe. Treatment is also exceptionally long–it takes 6 months in the simplest case and still carries a 15% risk of relapse after treatment. Specifically, we investigate different doses of rifampicin which is one of the key antibiotics in standard TB treatment. Higher-than-standard doses of rifampicin are potential strategy to shorten therapy duration.

We use mathematical models of rifampicin’s effect on bacteria and analyses of clinical trial datasets. We conclude that in rifampicin-treated TB, the likely cause for the observed slowdown is the heterogeneous elimination of different bacterial subpopulations within patients. In concordance with previous studies and the data analysis presented here, we predict that bacterial elimination can be accelerated by higher rifampicin doses.

## Introduction

Tuberculosis (TB) has been afflicting human populations for millennia. The standard treatment for antibiotic susceptible TB is a 6 month long combination of rifampicin, isoniazid, pyrazinamide, and ethambutol that still carries a significant risk of relapse: up to 15% [1,2]. Shortening these treatments is a public health priority; however, this is difficult to achieve due to the lack of insight into how antibiotic therapies affect bacterial populations *in vivo* in general, and especially in the case of TB.

In clinical trials of (pulmonary) TB, the clinical course of patients and treatment efficacy are often monitored with sputum mycobacterial cultures. In phase 2A of TB drug development, i.e. early bactericidal activity (EBA) trials, quantitative sputum bacterial counts are commonly used and measured daily for up to 14 days. Later phases of drug development focus on the subsequent time period in which sputum cultures return negative and therefore the time of sputum culture conversion from positive to negative (TSCC) is used instead [3,4]. TSCC has been shown to be correlated to the probability of relapse and unfavourable outcomes [5]. This indicates that the bacterial burden in sputum may be indicative of the total bacterial burden in a patient, even if the sputum alone does not allow a complete quantification of the bacterial burden. This is because i) not all bacteria in sputum can be cultured [6–8]; ii) only bacteria in (micro)cavities connected to the airways are thought to be detected in sputum, and intracellular bacteria or bacteria in granulomas remain inaccessible; iii) even though sputum culture conversion is expected to occur in the first 2 months of treatment, patients still have to be treated for the full 6 months in order to avoid relapse [1,2].

**Fig 1:**
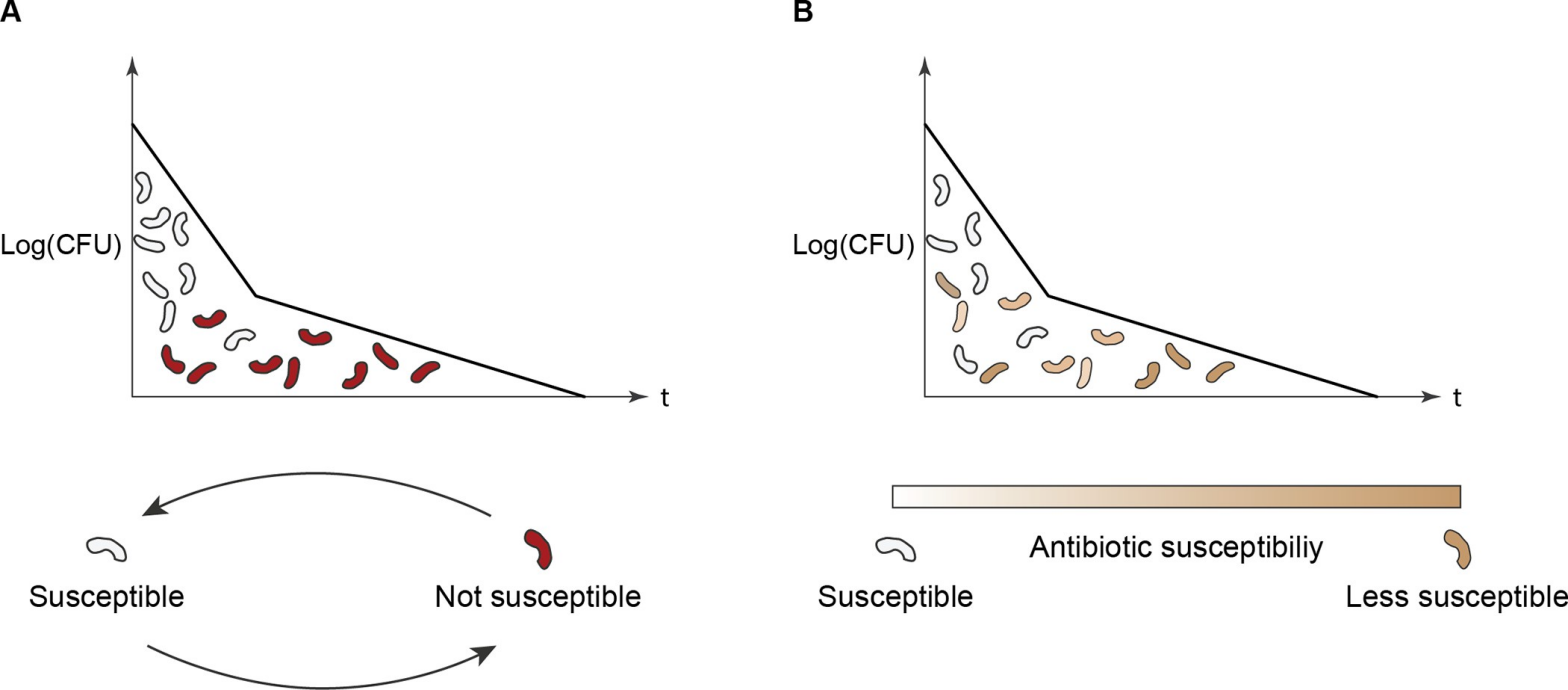
Mechanisms that can result in a slowdown in bacterial decline: persistence and heteroresistance. In both cases, X-axis is the time and the Y axis is the number of bacteria. **Fig 1A** illustrates persistence where bacteria switch back and forth between a susceptible and non-susceptible state over time only the non-susceptible ones remain. These are only killed when they exit a non-susceptible state. **Fig 1B** illustrates heteroresistance that is defined by a diversity within the susceptibility on antibiotics within the population of bacteria, some subpopulations will end up being killed at a slower pace.

In addition to imperfect quantification, the rate at which culturable bacteria in sputum decline is difficult to determine. This is because bacterial killing follows a typical biphasic pattern with a fast initial and a slow late decline. Such bi- or multiphasic bacterial killing has been observed both *in vivo* and *in vitro* across bacterial species [9–14]. The nature and cause of these biphasic kill curves are a hotly debated subject in microbiology. The different explanations for this slowdown can be divided into two general concepts: persistence (Fig 1A) and heteroresistance (Fig 1B) [11]. The concept of persistence implicates mechanisms that rely on switching back and forth between an antibiotic susceptible and a non-susceptible state. The most commonly shown mechanisms are the switch between a replicating and a non-replicating state, and a switch between intracellular and extracellular lifestyles [14–16]. The other concept, heteroresistance, implicates mechanisms that rely on diversity in the susceptibility to antibiotics within the population of bacteria [12,17,18]. While in TB research, heteroresistance usually refers to the coexistence of both susceptible and resistant strains, in microbiology it also refers to the observation that not all bacteria in a clonal culture exhibit the same antibiotic susceptibility [19]. Both phenomena have been shown to exist in *Mycobacterium tuberculosis* (*M*.*tb*) when exposed to rifampicin *in vitro* [20,21]. Both likely exist *in vivo* as well, although their clinical significance it is unknown [14,22,23]. Currently, it is unclear which mechanisms contribute to the biphasic decline of bacteria, including *M*.*tb* in sputum. This is difficult to investigate experimentally, because of the inaccessible location of the bacteria (most often lungs, but also other inaccessible organs) and because most microbiological characterizations require removing bacteria from the patients, thereby altering both the bacteria and their microenvironment.

Recently it was shown that the slow phase of bacterial killing may be predictive of treatment success, failure, or relapse [24]. Multiple other studies involving rifampicin, one key drug used in the treatment of TB, have linked bacterial load in sputum to treatment success: mathematical models based on clinical trials have linked rifampicin exposure to TSCC [25], as well as to the decrease in bacterial loads measured over 14 days [26]. Another study with a limited number of patients linked higher rifampicin doses per bodyweight to the rate of late bacterial decline [17]. Animal models have also predicted a significant treatment shortening with higher rifampicin concentrations [27]. However, the results of these studies are difficult to use in mathematical models that would aid in designing optimal TB therapy.

A quantitative approach that links antibiotic exposure to bacterial killing allows us to compare clinical results to mathematical model predictions and thereby aids in investigating the nature of the slowdown in bacterial decline. In mathematical models of heteroresistance, we have previously shown that the slow phase can only be affected by higher drug doses if it is caused by a diversity in decline rates, rather than switching between states [17,28]. However, we only investigated the effects of overall dose levels in [17] and did not address whether high intermittent (maximal Cmax) or lower constant time-concentration profiles are optimal for accelerating bacterial clearance. Here, we would like to investigate the effects of changing antibiotic time-concentration profiles in models of persistence and heteroresistance and compare them to observed patterns in patients with different pharmacokinetic characteristics. Depending on the causes behind the biphasic behaviour, pharmacokinetic parameters may have different impact on treatment success and bacterial counts.

In order to uncover the mechanisms behind the slowdown in bacterial decline, we analysed the bacterial count measurements from a clinical trial on varying doses of rifampicin in TB treatment (NCT01392911, [29]). Next, we compared the results from this data analysis with mathematical models of heteroresistance and persistence. Finally, based on the statistical analysis, we aimed to estimate how TSCC may depend on treatments and compare the estimates to measured TSCCs in a different clinical trial in TB treatment (NCT01785186, [30]). Our approach allows us to show not only the possible mechanisms behind the slowdown in elimination, but also how to mitigate them.

## Materials and methods

### Dataset

For the main analysis, we used the dataset of NCT01392911, an early bactericidal activity dose ranging trial on TB patients. Here, participants received 10, 20, 25, 30, 35 or 40 mg doses per kg bodyweight of rifampicin as a monotherapy for the initial seven days, after which isoniazid, pyrazinamide, and ethambutol were added in standard doses for the second seven days of the trial. The effects of this is discussed in the results. From this dataset, we only used the data of participants that had C_max_, AUC, as well as sputum bacterial count measurements available (n = 80). We only used AUC and C_max_ rather than PK measurements over time as the statistical analysis approach we used required us using a simple “one dimensional” PK driver to correlate our outcomes with. The AUC and Cmax values were optimal for this purpose.

The dataset contained both time to positivity measurements as well as CFU measurements to quantify bacterial burden. Only CFU counts were used as converting time to positivity to CFU measurements based on literature models would introduce additional challenges to the analysis. This is because TTP samples are based on measuring the time it takes for bacterial samples to reach certain densities through growth and thereby become positive samples. This positivity occurs around 10^5−6^ CFU/ml in the samples [31,32], as a result converting to CFU above these densities becomes a lot more uncertain and require non linear model [24,33]. This would have been difficult to correct and account for in our analysis.

CFU values below the limits of quantification were discarded as other methods for dealing with these datapoints would have introduced uncertainties to the analysis that would be difficult to account for in this exploratory work.

We estimated TSCCs (time to sputum culture conversion) using the main analysis and compared the estimates to the measured TSCCs in trial NCT01785186 [30]. This trial is a multi-arm multi-stage (MAMS) clinical trial on TB patients that included treatment arms on higher rifampicin doses. During the trial, the participants received 12 weeks of experimental treatments, followed by the standard continuation phase (rifampicin and isoniazid) treatment for another 14 weeks. Here, we only used data from the control (HRZE, standard regimen n = 123) and higher rifampicin dose standard regimen but with 35mg/kg rifampicin (HR35ZE, n = 63) treatment arms.

### Statistical analysis

In each patient, we had access to bacterial count determinations on 10 days. Our aim was to have at least 4 data points each for the early and late phase to estimate the rates of decline. Due to missing data, it was not possible to get reliable estimates of biphasic kill rates in substantial fraction of single patients. Therefore, we pooled bacterial counts from sputum samples from patients with similar pharmacokinetic measures. This also allowed us to include participants into the analysis that would not have enough measurements for fitting i.e. less than three days’ worth of measurements for the quick or the slow phase. We divided the range between the measured minimum and maximum pharmacokinetic measurements (either AUC or C_max_) into equal intervals. Next, using the least squares method, we fitted biphasic curves to the pooled Log (CFU) measurements from all patients within each of the intervals (see Eq (1)) for the fitted function). Finally, each of the intervals were assigned to have the median of the AUC and C_max_ values of the patients within. We used these in univariable analyses, where we tested the relationships between pharmacokinetic measures and the quick phase, slow phase, estimated last day, Log(CFU) on days 0 (both a subpopulation in the slow phase and total), day 14, and day of transition from quick phase to slow phase. Here, to estimate the last day of treatment, we extrapolated the fitted curves would go below 5CFU, based on the estimated detection limits as described in [34]. Log(CFU) on day 0 of a population in the slow phase was expected to stay above the limit of detection as the slow phases were estimated to be a decline in bacteria counts. For the relationships between the pharmacokinetic parameters and the quick or the slow phases we used weighted fits, where the weights were determined using the standard errors of the fits to the quick or to the slow phase (${\frac{1}{s.error}}^{2}$). This allowed us to give smaller weights to cases where we are more uncertain about our estimates. To account for multiple testing, we have corrected all the P-values using the Benjamini-Hochberg method.

$$B(t)=\left\{ \begin{array}{r} B(0)+d_{q}t,t<T\\ B(0)+d_{q}T+d_{s}(t-T),t\geq T \end{array}\right.$$ (1)

Finally, we used the corrected Akaike information criterion (AIC) values as well as the adjusted R-squared values of the fits assessing whether the C_max_ or the AUC is a better predictor for the slope of the slow phase.

#### Determining the day of transition from quick phase to slow phase

*In vivo* data is noisy, and the transition from quick phase to slow phase (based on mathematical models) individual fits of the biphasic curves. We have opted for determining the day of transition based on all the available fits rather than optimizing fits in for each biphasic curve individually. Therefore, we repeated the fitting procedure for all dosing groups with a different day of transition. We chose the day of transition where median R^2^ for the fits of the slow and fast decline were highest. We got the same result when minimizing the P-values as well.

### Modelling bacterial decline

#### PD models

For both of the models, we used the antibiotic concentration–net growth (decline) rate relationship available in the literature in [35] that processed data of *in vitro M*.*tb* exposure to rifampicin measurements by [36,37]:

$$\delta (A)=r-\frac{EC_{max}A}{EC_{50}+A}$$ (2)

where *A* is the antibiotic concentration in $\left[\frac{mg}{l}\right]$.

#### PK-PD models

The pharmacokinetic model used the published compartmental pharmacokinetic model of [40]. For the sake of simplicity, we only used absorption (Eq (3)), plasma (Eq (4), and tissue (Eq (5)) compartments and have not used a compartment chain for the absorption. Furthermore we have also omitted the time varying nature of rifampicin pharmacokinetics as the changes in parameters would have been unlikely to explain a slowdown in bacterial elimination [41], therefore choosing a published model that contains tissue distribution measurements was preferred [40]:

$$\frac{{dA}_{abs}}{dt}=input(t)-ka\;A_{abs}$$ (3)

$$\frac{{dA}_{plasma}}{dt}=ka\;A_{abs}-\frac{CL}{V}A_{plasma}$$ (4)

$$\frac{{dA}_{tissue}}{dt}=k_{pl-tissue}(R_{tissue}\frac{A_{plasma}}{V}-A_{tissue})$$ (5)

Here the parameters are:

- *input*(*t*) is the input function, to simulate daily doses of rifampicin. It implemented as Dirac delta functions (Dirac comb) spaced 24h apart.
- *A*_*abs*_, *A*_*tissue*_, and *A*_*plasma*_ are antibiotic concentrations in the absorption, plasma and tissue compartments
- *ka* = 1.55 [*h*^−1^]: absorption rates of the drug from absorption compartment
- $CL=5.72[\frac{L}{h}]$ are clearance rates of the drug from the plasma compartment
- *V* = 52.3 [*L*] is the volume of distribution in litres
- *R*_*tissue*_ = 069 is the penetration coefficient into the given tissue [40].
- *k*_*pl*−*tissue*_ = 1.68[*h*^−1^] rate of drug moving from plasma to tissue [40].

For the pharmacodynamic models, we omitted bacterial replication from the mathematical model for the sake of simplicity as well as the lack of data on the progeny of the less susceptible subpopulations as well as the persistent bacteria. Arguably there is very little replication taking place over the modelled time period (14 days). This is due to antibiotic concentrations being close to or above MIC for most of the time, post-antibiotic effect (lag time after antibiotic exposure before the replication restarts), and the slowness of replication in vivo environments. Here, the bacterial population sizes are calculated with Eq (13):

$$\frac{{dB}_{i}(t)}{dt}=\delta (A)B_{i}(t)$$ (6)

### Mathematical models of the slowdown in bacterial decline

An apparent slowdown in the decline rates can stem from a diversity in the decline rates within populations of bacteria or switching back and forth between susceptible and non-susceptible states. While the two are not mutually exclusive, one or the other may dominate the response on a given timescale. To model this, we use mathematical models of antibiotic persistence and heteroresistance in populations of bacteria in order to be able to assess the response of the two different mechanisms to different pharmacokinetic measurements. We are using these as a reference as both of these mechanisms has been observed in mycobacteria previously [12,42]. Table 1 summarizes the parameters and values used in the equations.

**Table 1:** Summary of parameters used in the mathematical models of persistence and heteroresistance.

Parameter	Description	Value	Unit	Ref
*EC*_*max*_	Maximum bacterial killing rate	4.32	*day* ^−1^	[35–38]
*EC*_50_	Half-maximal effective concentration	5.6	$\frac{mg}{l}$	[35–38]
r	Growth rate of *M*.*tb bacteria*	0.8	*day* ^−1^	[38,39]
*b*	Switch rate of *M*.*tb* from a non-replicating state to a replicating state	0.0016	*h* ^−1^	[14,15]
*B*_*total*_(0)	Total modelled population size	10^6^	-	Based on the dataset: B(0)_median_ = 10^5.9^ B(0)_mean_ = 10^6.11^

### Phenotypic switch

The most well-known example of switching back and forth between states is bacterial persistence caused by a dormant state [15]. Here, it is assumed that a subset of bacteria is in a non-replicating, dormant state that protects them from the effects of antibiotics that damage growing bacteria (for example rifampicin). Bacteria in their dormant state should be completely or highly tolerant to antibiotics, however once they switch back to a replicating state, they are swiftly killed by antibiotics. Mathematically this can be modelled as described in Eq (7) [15]. These equations describe the two subpopulations of bacteria, one that is susceptible (*n*) and one that is non-susceptible (*p*). Once the dormant state bacteria start replicating again, they become susceptible to antibiotics again (switching rate *b* in the equations).

$$\left\{ \begin{array}{c} \frac{dp}{dt}=-bp\\ \frac{dn}{dt}=bp+\delta (A)n \end{array}\right.$$ (7)

$$b=\frac{r_{M.tb}}{r_{E.coli}}b_{E.coli}$$ (8)

In [15], they have determined the switch rate (*b*) from non-replicating state to a replicating state for *E*.*coli* (persisters) to be. We have scaled this value by the differences in replication rates between *E*.*coli* and *M*.*tb* (Eq (8)) in order to preserve the assumptions of the original model on the determined fraction of persisters. This is supported by the literature [14] that found > 20 *day* lag times for *M*.*tb* (compared to our estimate of $\frac{1}{26}day^{-1}$ switch rates)

We chose to model the non-replicating or slowly replicating *M*.*tb* as non-susceptible to antibiotics, based on the current definition of persistence [11]. This is also supported by the literature that reported 17x, 50x, and 200x decrease in susceptibility to rifampicin in a non- or slowly replicating state [23,43,44]. This makes the decline rates of persisters negligibly small on the MIC ranges (1 – 16x MIC) we use the mathematical model on (S1 Fig shows the difference in having no decline in the model and persisters with 17x MIC which is on the higher end of estimates in the literature).

### Heteroresistance

The other mechanism responsible for the slowdown in the decline is heteroresistance [11,22]. The underlying assumption is that the bacteria in a population are not completely identical or may express unstable resistance genes that can result in a diversity in the susceptibility to external antibiotic concentrations. Additionally, there can also be a diversity in the expression of efflux pumps, targets, or cell sizes for example which can be described by the same model [12,17,18,28].

Mathematically, this can be calculated using Eq (9) [17,28] that describe multiple subsets of bacteria each with a slightly different susceptibility to antibiotics.

$$B_{total}(t)=\sum_{i}B_{i}(0)e^{\delta_{i}t}$$ (9)

here *Bi*(0) represents the initial size of the *i*-th subpopulation. Furthermore, *δ*_*i*_ are the corresponding decline rates.

To model a continuous distribution of resistant subpopulations with a normal distribution around a majority “average” susceptibility, we simulated 9 subpopulations where the middle one (5^th^) has the “average” susceptibility and in the majority.

$$B_{i}^{*}(0)=B_{total}(0)*D(i-5|0,1),where\;i=1\ldots 9$$ (10)

where *D*(*x*|*μ*, *σ*), is the probability density function of the normal distribution, with the mean: *μ* and standard deviation *σ* of the distribution. The number of subpopulations was decided based on estimating the distribution from the clinical trial data, assuming that there is a subpopulation with at least 8x MIC, up to 16x MIC to account for potential errors from the estimates. For computational reasons, we decided on 9 subpopulations to model the continuous distribution of resistances.

Due to the way this is calculated (by taking the value of the normal distribution at the given point), $\sum B_{i}^{*}$ will be less than *B*_*total*_ and therefore has to be rescaled:

$$B_{i}(0)=\frac{B_{total}(0)}{\sum B_{i}^{*}(0)}*B_{i}^{*}(0)$$ (11)

Here we simulate decrease susceptibility as changing the effective antibiotic concentrations (*A*_*i*_). For the purposes of this paper the two are the same, but this approach is easier to compute [28].

$$\delta_{i}=\delta (A_{i})$$ (12)

Therefore, we use the following to simulate 9 subpopulations with MICs ranging from 1/16^th^,1/8^th^… to 8x and 16x of the average:

$$A_{i}=(2^{i-5})A_{input}$$ (13)

This model was mainly used for simulations, that compares qualitative differences between persistence and heteroresistance, the parameters for the population distribution *D*(*x*|*μ*, *σ*) are the fits to the statistical analysis (*μ* = 0, *σ* = 1.3).

## Results

### Statistical analysis of the dataset

In this work we investigated possible mechanistic causes underlying the observed slowdown of bacterial decline rates in patients treated with different doses of rifampicin. We investigated the properties of the slowdown in elimination in response to different pharmacokinetic measures (total exposure in plasma or AUC; and peak plasma concentrations or Cmax) via statistical analysis, as different behaviours hint at the mechanistic causes of the slowdown. Here, a per-patient analysis is not viable, as fitting a distinct quick and slow phase to 10 days’ worth of data (4–6 points per phase) is unreliable due to the inherent noise of *in vivo* measurements. To resolve this, we pooled data together from multiple participants. In order to avoid bias from the choice of group sizes, we repeated the analysis with 30 different group sizes. We formed these groups by dividing the measured range of pharmacokinetic parameters (either Cmax or AUC) into 10 to 40 equal sized intervals. This ensured that a detected slowdown in elimination is consistent across patients with similar pharmacokinetic measurements, and the groupings do not introduce bias into the analysis. Unless otherwise indicated, we report the median values for all of the statistical descriptors (e.g. R^2^, p-value), and estimated parameters (reported in Table 2).

**Table 2:** Summary of the median fitted values to the dataset across all groupings, with day 4 as the day of transition from quick phase to slaw phase. All the P values reported are corrected for multiple testing with the Benjamini-Hochberg method. Here the overall decline rates (used in Eq (1)) within each phase can be calculated as: $d_{quick}(cMax)=intercept[day^{-1}]+slope[\frac{day^{-1}}{mg/L}]\cdot cMax[mg/L]$.

Property	Grouped by	Intercept (SE)	Slope (SE)	Intercept p-value (corrected for multiple testing)	Slope p-value (corrected for multiple testing)	R^2^ (adjusted)	AIC (corrected)
Quick [*day*^−1^]	AUC	-0.19 (±0.038)	-0.0005 (±0.00009)	0.0003	0.00007	0.67	-25
	C_max_	-0.1 (±0.057)	-0.0057 (±0.001)	0.1	0.00016	0.54	-22
Slow [*day*^−1^]	AUC	-0.075 (±0.024)	-0.00015 (±0.00005)	0.007	0.01	0.32	-43
	C_max_	-0.043 (±0.026)	-0.0018 (±0.00051)	0.12	0.003	0.34	-57
Baseline Log(CFU) [–]	AUC	5.54 (±0.018)	0.0012 (±0.00044)	10^−15^	0.0124	0.30	23
	C_max_	5.27 (±0.254)	0.016 (±0.0052)	10^−13^	0.0107	0.27	40
4^th^ day Log(CFU) [–]	AUC	4.77 (±0.26)	-0.0009 (±0.00063)	10^−11^	0.163	0.30	23
	C_max_	4.86 (±0.307)	-0.008 (±0.0064)	10^−12^	0.23	0.27	40

For each groups’ pooled sputum bacterial count measurements (for all groupings), we fitted biphasic curves with different days of transition from the quick killing to the slow killing of the bacteria. We determined the most probable days of transition, day 3 or 4, based on the median R^2^ (see S2 Fig) and median p-values of the fits (see **S3 Fig)**. For the rest of the analyses, we used day 4 as the day of transition between the quick and the slow phase.

**Fig 2:**
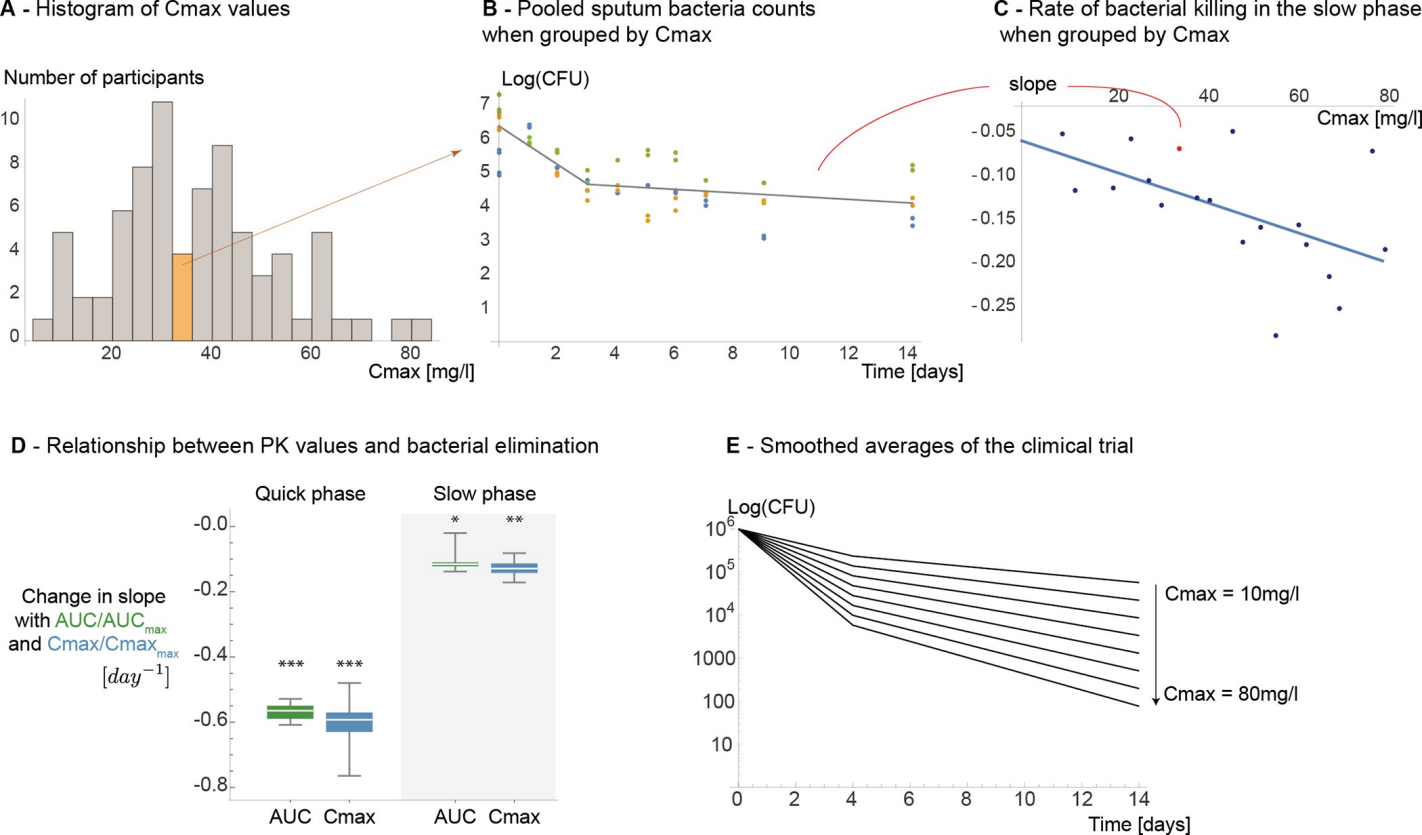
Overview of the fitting process and the relationship between the decline rates of bacteria and pharmacokinetic measures. We have grouped participants (**Fig 2A**) by forming equal groups within the pharmacokinetic measures. For each group, we pooled the bacterial count measurements (Y-axis) of all participants (dots, here one colour corresponds to one participant) and fitted biphasic curves to them (solid line) (**Fig 2B**). Next, we analysed the relationship between the groups’ median pharmacokinetic measurements (X axis) and the properties of the fitted curves (Y axis) (**Fig 2C**). **Fig 2D** summarizes the dependence of the decline rates in the quick and slow phases on $\frac{AUC}{AUC_{max}}$ and $\frac{Cmax}{Cmax_{max}}$. Here, we have normalized the values (by dividing with the maximum), in order to be able to show that both measures produce the same result but with different errors. **Fig 2E** demonstrates how this affects the bacterial count measurements (X-axis) over time (Y-axis). The curves were made with Eq (1), starting at 10^6^ CFU/ml bacteria, using day 4 as the day of transition, with the obtained fits for decline rates (see Table 1), and Cmax values of 10, 20, 30…80 [mg/l] (in the dataset the Cmax values range from 7.7 to 85.6 mg/ml).

Based on the fitted curves, we found that both phases of decline are dependent on antibiotic concentrations and therefore pharmacokinetic measures. This is demonstrated in Fig 2 which illustrates the dependence of kill rates on AUC or Cmax_._ The resulting smoothed time-kill curves based on the fits are shown in Fig 2E. Table 2 summarizes the properties of the fitted curves, S4 Fig shows all the determined fits for all different transition days, and S3 Fig shows the corresponding p-values. We applied the Benjamini-Hochberg correction to account for multiple hypothesis testing when calculating all p-values reported in Table 2 and **S3 Fig**.

### Mathematical modelling of heteroresistance and persistence

**Fig 3:**
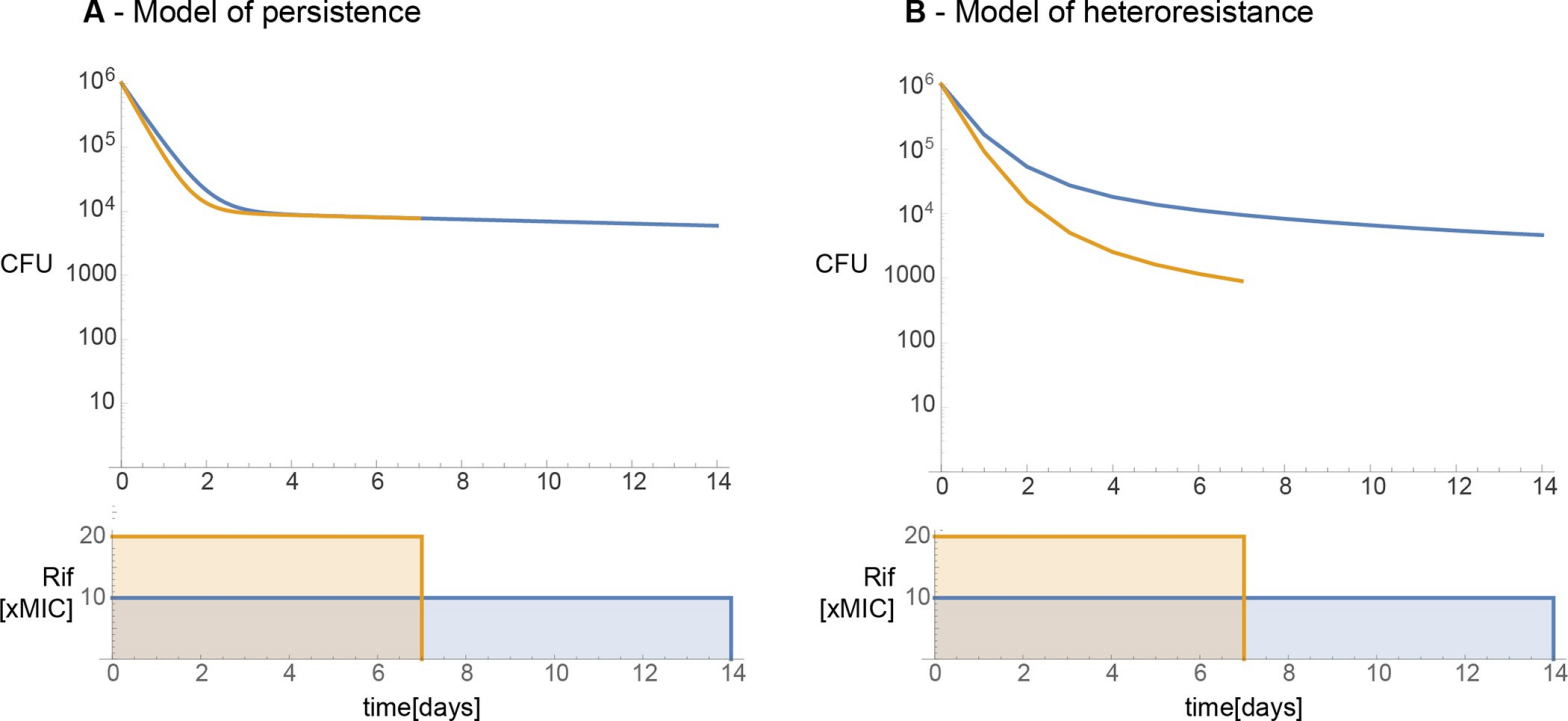
Predicted responses of the mathematical models of persistence and heteroresistance. This figure shows the qualitative difference between the causes behind the slowdown in bacterial decline. The top row shows bacterial counts (Y-axis) over time (X-axis) for two different idealized dosing regimens (orange and blue curves). The bottom row shows the two simplified exposure profiles: both have the same AUC but different Cmax values. **Fig 3A** shows that if the slowdown in decline is a result of switching between susceptible and non-susceptible states, then the slope of the slow phase is not dependent on C_max_. On the other hand, **Fig 3B** shows that if the slowdown in decline is the result of a diversity in the susceptibility to antibiotics, then a change in antibiotic concentrations should affect the slope of the slow phase as well.

We compared the predictive power of the pharmacokinetic measures using the adjusted R2 (S2 Fig) and corrected Akaike Information Criterion (AIC) values (S5 Fig) of the fits. The quick phase was better predicted by the AUC rather than the Cmax, while the slow phase was better predicted by the Cmax rather than the AUC. The dependence on pharmacokinetic measures of the quick phase was expected as rifampicin efficacy is thought to be better predicted by the AUC [45]. However, the slow phase’s dependence on Cmax was previously unknown and can both aid in optimizing therapy and give insight into the possible mechanistic causes of the slowdown. We demonstrate this in Fig 3 in which we modelled the responses of the two main possible causes of the slowdown (persistence and heteroresistance) when *M*.*tb* is exposed to rifampicin. We used simplified exposure profiles in which the AUC remains constant: a sustained high concentration (Cmax) for shorter duration of time and a low sustained concentration for longer duration of time. We found that heteroresistance predicts that antibiotic concentrations affect the slope of the slow phase, while persistence predicts that antibiotic concentrations will have no effect on the slope of the slow phase. The simplified cases also demonstrate that a high Cmax is required to kill the less susceptible subpopulations in heteroresistance. Therefore, our observations on the properties of the slow phase are consistent with the definitions and models of heteroresistance and are inconsistent with the definition and mathematical models of persistence [11].

**Fig 4:**
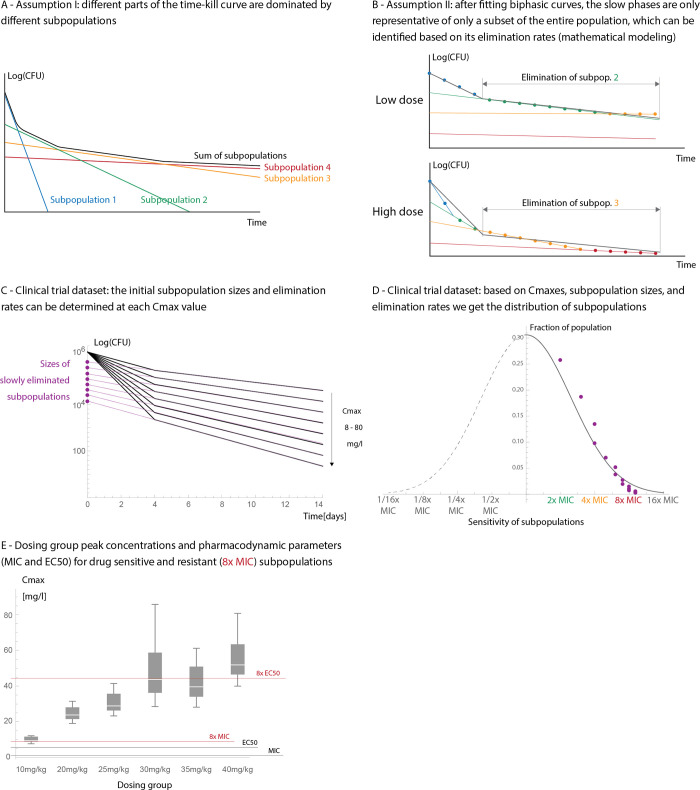
Estimating the sensitivity distribution of bacteria. **This figure shows the method we used to** estimate the sensitivity distribution of bacteria when the slowdown is caused by heteroresistance. **Fig 4A (illustration)** shows that different parts of the time-kill curves (bacterial count (Y-axis) over time (X-axis)) represent the decline of subpopulations with different MICs (different colours) [28]. In these cases, even after fitting biphasic curves on a multi-phasic time-kill curves (**Fig 4B (illustration))**, the slow phase will represent a subset of subpopulations which can be identified based on the decline rates at the given antibiotic concentration (Cmax or AUC in case of PK based models). **Fig 4C** shows the parameters used obtained from clinical trial dataset: decline rates in the slow phase and the corresponding “subpopulation sizes” at each Cmax. **Fig 4D** shows the value pairs from **Fig 4C**: the obtained sensitivity distribution and a fitted (assumed) normal distribution to it. **Fig 4E** shows the distribution of Cmax values in each dosing group and how they compare to the pharmacodynamic parameters of MIC (1.3mg/l) and EC50 (5.6mg/l, concentration at which bactericidal activity is 50%) [35]. These values are marked for both a drug sensitive subpopulations and a less susceptible subpopulation (8x MIC) to illustrate how the sensitivity of bacterial subpopulations in Fig 4D relate to the pharmacodynamic parameters. Here, we assume that the EC50 values of the less susceptible (8x MIC) subpopulations are 8x the original EC50 values. However, the correct relationship between MIC and EC50 in different subpopulations is unknown as resistance mutations have a chance of affecting the slope of the PD curves not just the effective antibiotic concentration [46], therefore this figure is for illustrative purposes.

Heteroresistance is defined as the coexistence of multiple subpopulations with different susceptibilities, where some (minority) subpopulations have at least 4 or 8x the MIC when compared to the majority of the population [10,11]. Therefore, to further assess whether the slowdown in decline is caused by heteroresistance we calculated the hypothetical sensitivity distribution of bacteria based on the observed slowdown. Fig 4A and 4B illustrate the approach, which is based on combining PK-PD modelling with the results of the statistical analysis of the clinical trial. We calculated how subpopulations with different sensitivities in cavity walls would be killed with daily doses of rifampicin and compared this to the decline rates of the slow phase in the clinical trial at each level of Cmax. This allowed us to derive the sensitivity of the subpopulation that dominates the slow phase. We assigned the corresponding subpopulation size (based on the dataset Fig 4C) to each subpopulation that yielded the sensitivity distribution of bacteria (Fig 4D, purple dots). The resulting distribution shows a strong agreement with the definitions of heteroresistance (a distribution of resistances, with at least 4-8x MIC subpopulations). In persistence, the size of the subpopulation with a slow decline should be independent of the antibiotic concentrations (i.e. Fig 4C would show different initial subpopulation sizes and the late bacterial decline rates would be indistinguishable).

**Fig 5:**
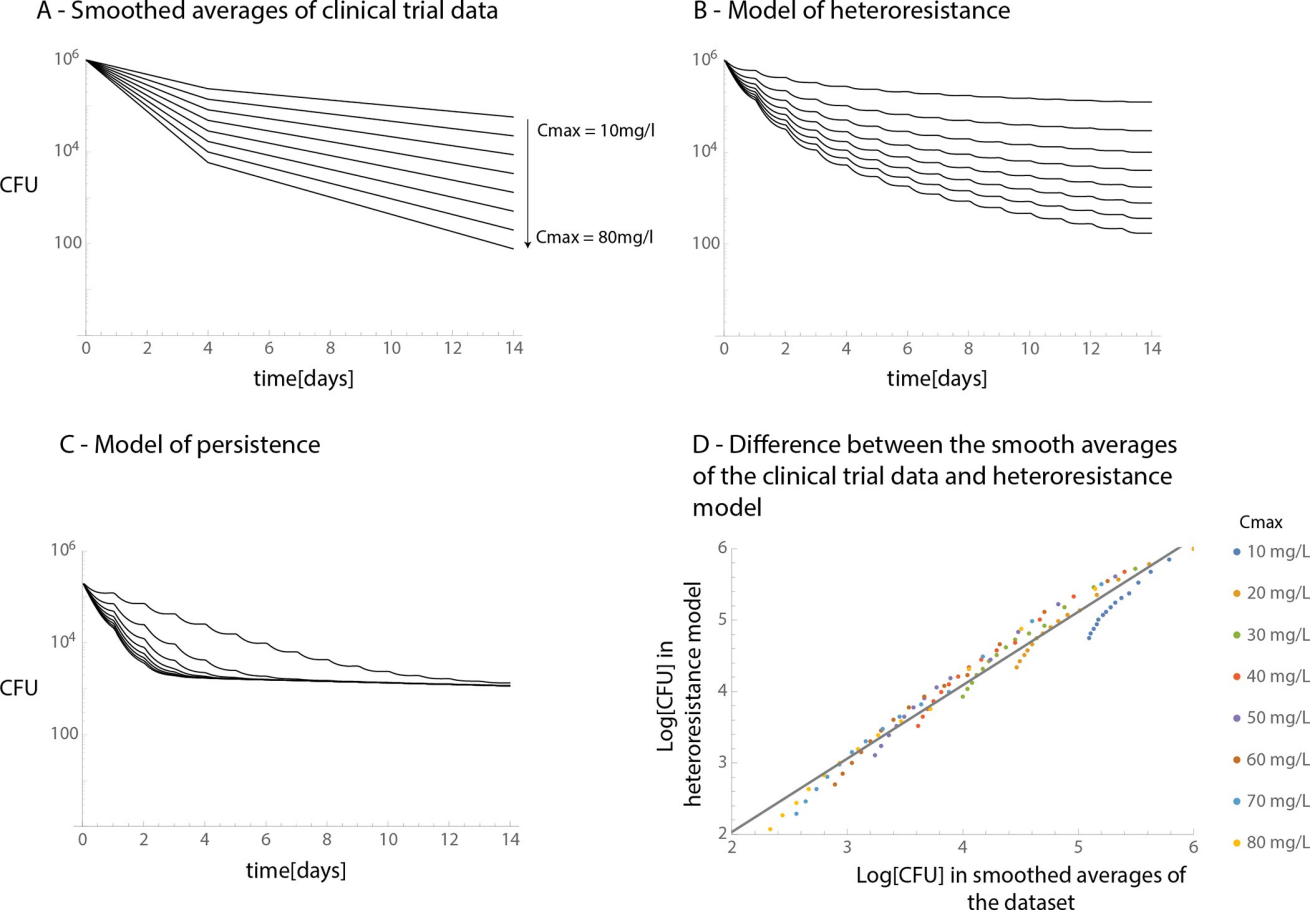
Comparison of the smoothed averages of the clinical trial and model predictions on sputum bacterial counts over time. This figure compares the smoothed averages of the clinical trial (**Fig 5A,** this is the same plot as in Fig 2E), the predicted decline of bacteria in cavity walls if the slowdown is caused by heteroresistance (**Fig 5B**) or persistence (**Fig 5C**). We generated these plots using pharmacokinetic models in the cavity wall with the same Cmax values as in Fig 5A (10…80 mg/l). Finally, **Fig 5D** shows the difference between the curves on Fig 5A and 5B: on all days and Cmax values combined, the X axis shows the model predictions and the Y axis shows the smoothed averages of the clinical trial ($R_{adj}^{2}=0.97$).

We also compared how well the PK-PD model of heteroresistance predicts the observed slowdown in decline when compared to the smoothed averages of the clinical trial dataset. To do so, we used the same model as for determining the sensitivity distributions. The parameters of the mathematical model are based on the literature: the pharmacokinetic model is based on [40] and the pharmacodynamic parameters on [35]. Fig 4 shows the sensitivity distribution in heteroresistance, which is fitted to the clinical trial dataset (assumed Gaussian distribution, *σ* = 1.3 truncated at 16x MIC, see black line Fig 4D). Fig 5A, 5B and 5C show side by side the decline rates of bacteria in the clinical trial, as well as the predicted decline rates by heteroresistance and for persistence. Fig 5D demonstrates that when compared, the difference between the observed decline of bacteria in the clinical trial and the one predicted by heteroresistance stay within one order of magnitude (see S6 Fig for individual comparison of the curves, note that Fig 5D is intended to assist in the comparison of the curves rather than serve as a diagnostic plot). Therefore, we conclude that this model can sufficiently explain the observed slowdown on this timescale.

Within dosing groups, C_max_ varied substantially from individual to individual meaning that even if the average patient reaches the threshold for eliminating 8x MIC subpopulations, others may not. Fig 4E demonstrates this, there we plotted the relationship between the observed peak drug concentrations per dosing group and marking the pharmacodynamic parameters of MIC and EC50. The latter, EC50 is the concentration at which the elimination rate of bacteria is 50% of the maximum. This shows that for the 10mg/kg group, even in the plasma, the 8x MIC may not be reached. Concentrations at which bacterial elimination of a less susceptible subpopulation is “efficiently eliminated (i.e. at the peaks there is briefly 50% the maximum elimination rate)” may not be reached in the dosing groups below 30mg/kg.

### Estimating the time to sputum culture conversion

**Fig 6:**
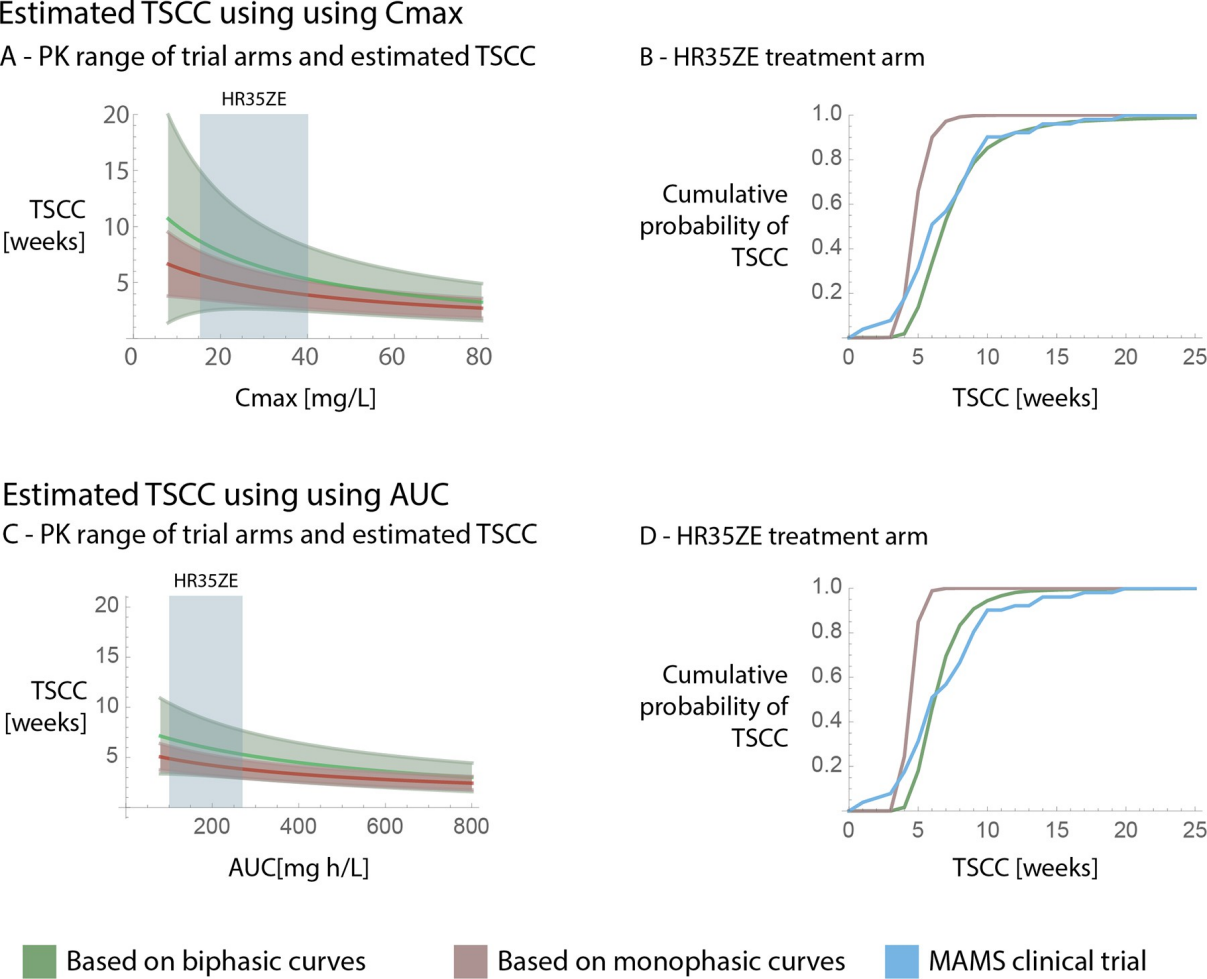
Estimated TSCC based on the statistical analysis, and comparison to measured TSCCs in a multi arm multi stage (MAMS) clinical trial (NCT01785186, [30]). Here, the green curves show estimates based on biphasic curves (i.e taking the slowdown in decline into account), while red shows estimation based on monophasic curves (i.e. neglecting the possibility of a slowdown in decline). Blue always corresponds to data from the MAMS clinical trial. Fig 5A and 5C show the dependence of the predicted TSCC on the pharmacokinetic parameters (Cmax and AUC respectively), as well as the measured pharmacokinetic ranges for the HR35ZE treatment arm of the MAMS clinical trial used for comparison (blue boxes). Here, the area around the estimates signify the 95% confidence interval around our estimates. The estimates are only shown within the parameter ranges of the EBA clinical trial. Fig 5B and 5D show the cumulative probability (Y-axis) of TSCC (X-axis) based on the estimates within the PK ranges of the MAMS trial as well as the data from the MAMS trial itself.

Finally, we estimated the expected time to sputum culture conversion based on the statistical analysis, assuming that there is no additional slowdown outside the timeframe of the clinical trial. Fig 6 shows the expected time to culture conversion depending on Cmax and AUC. We repeated the statistical analysis using monophasic kill curves (i.e. neglecting the possibility of a slowdown in bacterial decline (see S1 Table)) and estimated TSCC that way as well. We compared both estimates to the measured times to sputum culture conversion of a multi-arm multi-stage (MAMS) clinical trial on higher rifampicin doses (NCT01785186, [30]). From the MAMS trial, we used the higher rifampicin standard treatment with an increased, 35mg/kg rifampicin dose arm (HR35ZE). Estimates that take the slowdown in bacterial decline into account (biphasic rather than monophasic fits) provide estimates that are closer to the observed data and are more conservative. In addition we also compared estimates based on the standard dosing (HRZE, standard treatment with 10mg/kg rifampicin) treatment arm. There, the Cmax and AUC range of the MAMS trials were outside the range of the Cmax and AUC values of the EBA trial we fitted the model to. Therefore, these comparisons are provided for completeness’ sake and can be found in the supplement (S7 Fig). For the HR35ZE treatment arms, predictions of TSCC do not differ significantly between Cmax and AUC as in both cases concentrations are well above the MICs for most subpopulations in most of the anatomical sites and therefore are expected to be similar.

## Discussion

We analysed the measured sputum colony counts in a rifampicin dose ranging measuring early bactericidal activity (EBA) and found that there is a slowdown in the bacterial killing rates, resulting in distinct quick (days 1–4) and slow phases (days 5–14). These phases correlated significantly with patient rifampicin plasma concentrations. In case of the slow phase, this was previously unclear as the dependence on pharmacokinetic parameters of the slow phase can depend on the mechanism causing the slowdown. Therefore, it may or may not respond to increased antibiotic concentrations. Furthermore, we found that in the slow phase, the peak drug concentration (Cmax) is a better predictor for bacterial killing than the overall exposure (AUC). We have done this by showing a direct statistical link between measured pharmacokinetic parameters and sputum colony counts at different stages of decline.

This analysis of the dataset can be used to differentiate between the mechanistic causes of the slowdown. In microbiology, there are two main explanations for a slowdown in decline of bacteria: antibiotic persistence and heteroresistance [11]. Both persistence and heteroresistance have been consistently shown to exist even in cultures of clonal bacterial populations, including *M*.*tb* [12,14,42,47]. Additionally, both are not mutually exclusive, can act on different timescales and are dependent on the mode of actions of the antibiotics used. While the clinical significances of both phenomena are unknown [9–11], both have been shown to exist in in TB patients [13,14,20,22,42,48]. Most recently, differences in rifampicin response in TB patients was linked to heterogeneity in the bacterial population using NGS, however the mechanism (heteroresistance or persistence) was not described [49]. In persistence, the slow phase is driven by bacteria exiting a non-susceptible state which is commonly assumed to be independent of antibiotic concentrations. Therefore, higher antibiotic concentrations would not accelerate the slow phase of decline to the extent observed in the dataset [11]. In heteroresistance, the slow phase is driven by subpopulations that are less susceptible to antibiotics than the majority of the bacterial population. Therefore, higher antibiotic concentrations would accelerate the slow phase decline similarly to the dependence observed in the dataset [10,11,17,28]. The findings from the data analysis are corroborated by the concentration dependences observed by others as well [34]. Our statistical analysis has shown that the results are only compatible with the definitions of heteroresistance and not persistence.

To validate the findings based on the analysis of the dataset we compared the results to mathematical models of both persistence and heteroresistance parametrized with data available in the literature. The pharmacodynamics component is based on *in vitro* data of *M*.*tb* exposed to rifampicin [35–38]. This meant that for instance we used the maximum possible replication rates under lab conditions which is unlikely to be same as *in vivo* replication rates [50]. Changing the replication rates in the model would have shifted the balance of bacterial replication and elimination in the model and thereby the MIC. This would have introduced unknowns when it came to using the model of persistence (i.e. non-replicating bacteria). Furthermore, as the “true” *in vivo* MIC is unknown, changing replication rates that affect this parameter would have been misleading. As a result we opted to use an *in vitro* model with its original parameters. The pharmacokinetic model (where applicable) is based on models of rifampicin tissue concentrations in TB patients [40]. For the simulations we used cavity walls as open cavities are thought to be one of the sources of sputum. We found that the most probable cause of the slowdown in decline on this time scale (in response to rifampicin) is heteroresistance rather than antibiotic persistence. An alternative hypothesis to this is that the slowdown in bacterial decline is caused by the heterogenous distribution of drugs in the lungs that results in diverse decline rates in bacteria [13,51,52]. In our case, this scenario is unlikely, as our results indicate that some subpopulations are likely to have 8x the MIC meaning that some tissues would have 1/8^th^ of the drug concentration. The variation in exposure levels in different tissues cannot explain this large difference [40]. Additionally, the different environments in different anatomical sites could result in different susceptibilities to antibiotics as well [13], however there are no available pharmacodynamic models on this that could be used for modelling. Finally, rifampicin pharmacokinetics has shown to be nonlinear over time (and dose) [41]. Similarly to the case of heterogeneous distribution of antibiotics in tissues, it is unlikely to be solely accountable for the observed slowdown in elimination, however it may still be a confounder. As these are all possibilities, a more cautious summary of our results is that the slowdown in elimination is caused by heterogeneous bacterial elimination rates within the compartments from which the sputum is originating.

Through mathematical modelling we also show that the observed PK dependence of the quick and the slow phases are consistent with heteroresistance. In heteroresistance, the quick phase represents the elimination of the majority of the bacterial populations, where of the decline should be better predicted by the AUC than Cmax due to the AUC dependence of rifampicin [45]. In the slow phase however, the Cmax should be a better predictor than the AUC (S5 Fig). This is because the slow phase represents the decline of the less susceptible subpopulations where an increased MIC has to be overcome. Increasing AUC can potentially decrease Cmax which in turn may reduce the ability to overcome the higher MICs of some subpopulations. This is also what we have found in the dataset where the quick phase showed a stronger relationship with the AUC while the slow phase showed a stronger relationship with the Cmax. These differences were observable despite the fact that only one drug formulation and dosing regimen was used in the clinical trial and therefore the Cmax and AUC measurements are highly correlated. Consequently, the analysis on the differences between the effects of Cmax and AUC rely on the patient-to-patient variance in the drug absorption, elimination, and distribution rates. We expect the differences between Cmax and AUC to be more pronounced in trials that vary drug formulations or dosing strategies as well as drug doses. This highlights the possibility of further optimizing treatments and are in support of high rifampicin peak concentrations. The current higher dose rifapentine trials (NCT02410772 [53]) can provide more clues on optimization once they are published as rifapentine has a different pharmacokinetic profile than rifampicin. However, it is difficult to predict which type of time-concentration profile would be ideal. First, it is unclear whether the patient’s tolerability depends on AUC or Cmax. Second, it has been argued that antibiotics used in TB treatments should be matched with others that have similar half-lives to avoid only one drug being present (functional-monotherapy [40]) and thereby not facilitating the emergence of resistance.

The clinical significance of heteroresistance is still unknown [9,10], however we demonstrate that a slowdown in bacterial decline due to heteroresistance delays the time to sputum culture conversion (TSCC) and therefore may reduce treatment success rates. An earlier TSCC is mildly associated with treatment success and the lack of it is associated with unfavourable outcomes and are important biomarkers in TB research [5,54]. We estimated TSCC based on the statistical analysis that assumes that there is a slowdown in bacterial decline, then we repeated the analyses by assuming that there is no slowdown (i.e. fitting a straight line to the bacterial counts) as a control. Next, we compared these estimates to the measured TSCCs from a multi arm multi stage (MAMS) clinical trial on high rifampicin doses. We show that with the use of biphasic curves the estimates on TSCC are not only more conservative but show better agreement with the data used for comparison. This validates our analysis and shows that the slowdown in elimination is present during treatments. It also serves as a demonstration on the impact of neglecting the possibility of slowdown on assessing experimental treatments. In concordance with the recently published paper using data from the first 8 weeks of treatment, our observations based on the first two weeks imply that the decline rate may not change significantly between 2–8 weeks. These results argue against the practice of having more sparse bacteriological assessments in the second half EBA clinical trials (as it was done in this trial as well), because it makes investigating the possible slowdown in bacterial decline more difficult.

These findings strengthen the connection between quantitative bacteriological assessments in EBA trials (sputum bacterial count measurements) and the TSCC that is used in subsequent phases of trials. The importance of strengthening this connection has been highlighted in the recent literature as one of the current challenges in TB clinical trial design [3,4]. We also identify a possible source of errors when evaluating EBA trials: not all drugs or drug combinations may result in the same slowdown in bacterial killing. Additionally, a slowdown may be outside of the timeframe of the two weeks of the trial (for example in bedaquiline [55]), causing us to significantly underestimate TSCC. Conversely, some drug combinations may cause little to no slowdown–in EBA trials, these may appear inferior to other treatment arms that have a fast initial decline of bacteria and a slowdown later on.

This work has several limitations as well: first, due to the number of participants in the study it was not possible to correct for the observed association between the baseline bacterial loads and the PK values. While it does not affect the calculation of decline rates itself, it may be a confounder [56,57]. Second, in the EBA trial during the first week, all the participants received rifampicin monotherapy; in the second week they have also received the standard doses of the remaining TB drugs (isoniazid, pyrazinamide, ethambutol) in combination with rifampicin. We have seen no changes in the dataset after day 7. Additionally, in the MAMS trial as all groups received the combination therapy from the first day, however there was still an agreement between the estimates based on the EBA trial and the MAMS trial. Therefore, the inclusion of the other TB drugs used for drug susceptible TB should not affect our conclusions substantially, however this cannot be ruled out. Furthermore, the inclusion of the other drugs makes it difficult to assess whether our results are true for rifampicin monotherapy by itself or just for combination therapy. Third, the PK PD model of heteroresistance currently cannot be used to predict TSCC. This is because in the model there are some subpopulations with very low susceptibility (16x MIC compared to the majority of the population) that are killed extremely slowly by antibiotics. These differences between the model and measurements can be due to the following: (i) the real susceptibility distribution of bacteria may be different, and subpopulations with very low susceptibility may not exist. (ii) In the model, the less susceptible subpopulations are modelled as having decreased effective antibiotic concentrations [46]. (iii) Immune system mediated killing of bacteria is not modelled as the killing rates for it are unknown. However, there is evidence that the immune system also plays a role in controlling TB infections [58–61]. Omitting these from the PK PD models can affect the estimated TSCC in various ways and may correct for an overestimated TSCC when accounting for subpopulation with >16MIC. This would imply that the immune system by itself may be able to handle very small bacterial populations. Fourth, bacterial colony counts in solid media only reflect culturable bacteria, however not all bacteria in a sample can be cultured [6–8]. Antibiotic exposure (including rifampicin) can increase the fraction of the non-culturable subpopulation [62,63]. The observed decrease in CFU with higher doses of rifampicin can be confounded by the increased fraction in non-culturable cells. Accounting for this confounder was out of the scope of this analysis.

Taken together, our results suggest that the bactericidal activity of TB treatments can be enhanced by higher rifampicin doses as well as by optimizing for treatment strategies and rifampicin formulations that allow peak concentrations above the MIC of all subpopulations of bacteria. This in turn may increase treatment success rates by reducing the time to sputum culture conversion: the time until no more bacteria are detected in the sputum. A shorter time to sputum conversion has been shown to be mildly correlated with treatment success [5]. This is supported by previous works on the same trial, which linked higher rifampicin exposures to an increased probability of earlier culture conversion [26], and increased time to positivity in liquid cultures [25]. Other studies have also shown that higher doses of rifampicin shorten treatment durations in mouse models of both *M*.*tb* [27] and *Mycobacterium ulcerans* [64]. Finally, with a limited number of patients, in a different study we linked higher rifampicin doses per bodyweight to the rate of late killing [17]. Therefore, our work adds to the growing body of literature supporting optimized rifampicin doses for TB therapy and give hope that optimized higher doses could allow shortening treatment as is currently investigated in ongoing clinical trials (NCT02581527, NCT02410772, [29,53,64]).

## Supporting information

S1 Fig Predicted responses of the mathematical models of persistence. **S1A Fig is the same as Fig 3A in the main text.** This figure shows the negligible difference between excluding (S1A Fig) and including elimination of persisters (S1B Fig). In the latter case persisters are modelled as a subpopulation with high, 17x MIC (one of the higher estimates in the literature [43]). The top row shows bacterial counts (Y-axis) over time (X-axis) for two different idealized dosing regimens (orange and blue curves). The bottom row shows the two simplified exposure profiles: both have the same AUC but different Cmax values.
(TIF)

S2 Fig Adjusted R squared values for the fits on the quick and slow phases’ dependence on PK values. The different box-whisker plots in each figure correspond to a different set day of transition. These plots both show that values are consistently better predictors for the slope of the slow phase (based on adjusted R-squared values), as well as that we achieve the best fits for days 3 and 4.
(TIF)

S3 Fig P-values for the fitted data with different day of transition (days 2–7, Y-axis) from quick to slow phase. The box-whisker plots represent the results from groupings from dividing the range of PK values into 10–40 equal intervals. These plots show that if we get the best fits if the days of transition are set to day 3 or 4. Furthermore these also show that C_max_ is consistently a better predictor for the slope of the slow phase than the AUC.
(TIF)

S4 Fig The determined fits of the biphasic curves for different days of transition (days 2–7, Y-axis) from quick to slow phase. The box-whisker plots represent the results from groupings from dividing the range of PK values into 10–40 equal intervals.
(TIF)

S5 Fig Difference of corrected AIC values for the fits on the slow and quick phases for different days of transition. Here, the positive values indicate that Cmax is a better predictor, while negative values would indicate that AUC is a better predictor for the given phase.
(TIF)

S6 Fig Side by side comparison of the smoothed averages of the clinical trial dataset and the mathematical model of heteroresistance. All figures show the observed/predicted bacterial counts (Y-axis) over time (X-axis). Each figure shows the same at different Cmaxes. The C_max_-es were chosen to be at regular intervals within the range of the clinical trial dataset (10–80 mg/l), the inputs doses for the mathematical models were chosen to achieve the same Cmaxes within the model.
(TIF)

S7 Fig Estimated TSCC based on the statistical analysis, and comparison to measured TSCCs in a multi arm multi stage (MAMS) clinical trial (NCT01785186, [30]). Note: this the left and right columns of this figure are the same as Fig 6. but extended with the standard dosing group for comparison. There, estimates relied on extrapolation outside the range of PK values of the EBA trials (see S7A and S7D Fig). On all of the plots the green curves show estimates based on biphasic curves (i.e taking the slowdown in decline into account), while red shows estimation based on monophasic curves (i.e. neglecting the possibility of a slowdown in decline). Blue always corresponds to data from the MAMS clinical trial. **S7A** and **S7D Fig** show the dependence of the predicted TSCC on the pharmacokinetic parameters (Cmax and AUC respectively), as well as the measured pharmacokinetic ranges for the HRZE and HR35ZE treatment arms in the MAMS clinical trial used for comparison (blue boxes). Here, the area around the estimates signify the 95% confidence interval around our estimates. The estimates are only shown within the parameter ranges of the EBA clinical trial. **S7B, S7C, S7E,** and **S7FF** Fig show the cumulative probability (Y-axis) of TSCC (X-axis) based on the estimates within the PK ranges of the MAMS trial as well as the data from the MAMS trial itself. This is shown for both Cmax and AUC, as well as the HRZE, and HR35ZE treatment arms.
(TIF)

S1 Table Summary of the median fitted values to the dataset across all groupings, when assuming a monophasic killing (fitting a straight line though the data). All the P values reported are corrected for multiple testing with the Benjamini-Hochberg method.
(DOCX)
