# Supplementary figures and images for: High rifampicin peak plasma concentrations accelerate the slow phase of bacterial decline in tuberculosis patients: Evidence for heteroresistance
Source: PLoS Comput Biol. 2023 Apr 13;19(4):e1011000. doi: 10.1371/journal.pcbi.1011000 (PMC10128972; doi:10.1371/journal.pcbi.1011000)

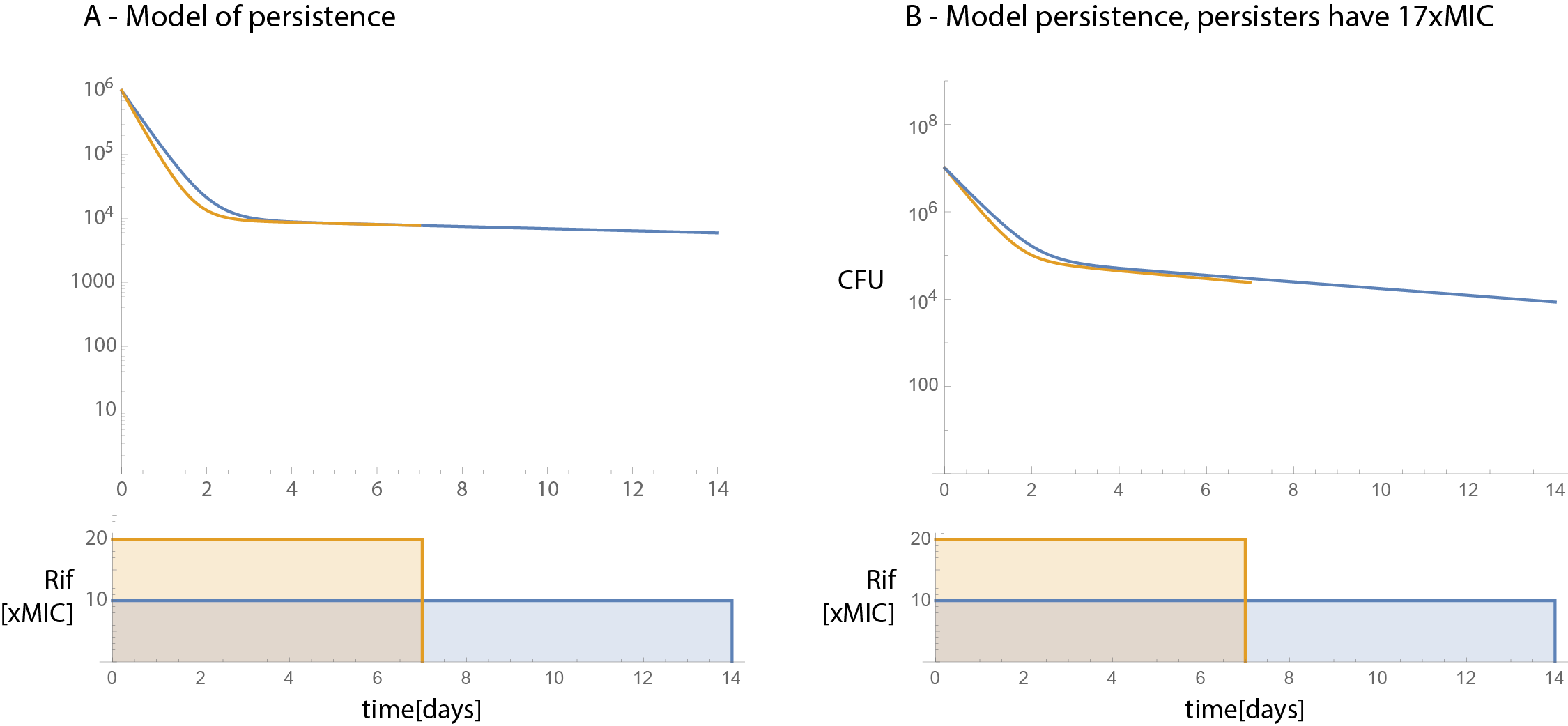

Supplement: S1 Fig — S1A Fig is the same as Fig 3A in the main text. This figure shows the negligible difference between excluding (S1A Fig) and including elimination of persisters (S1B Fig). In the latter case persisters are modelled as a subpopulation with high, 17x MIC (one of the higher estimates in the literature [43]). The top row shows bacterial counts (Y-axis) over time (X-axis) for two different idealized dosing regimens (orange and blue curves). The bottom row shows the two simplified exposure profiles: both have the same AUC but different Cmax values. (TIF) [file pcbi.1011000.s001.tif]

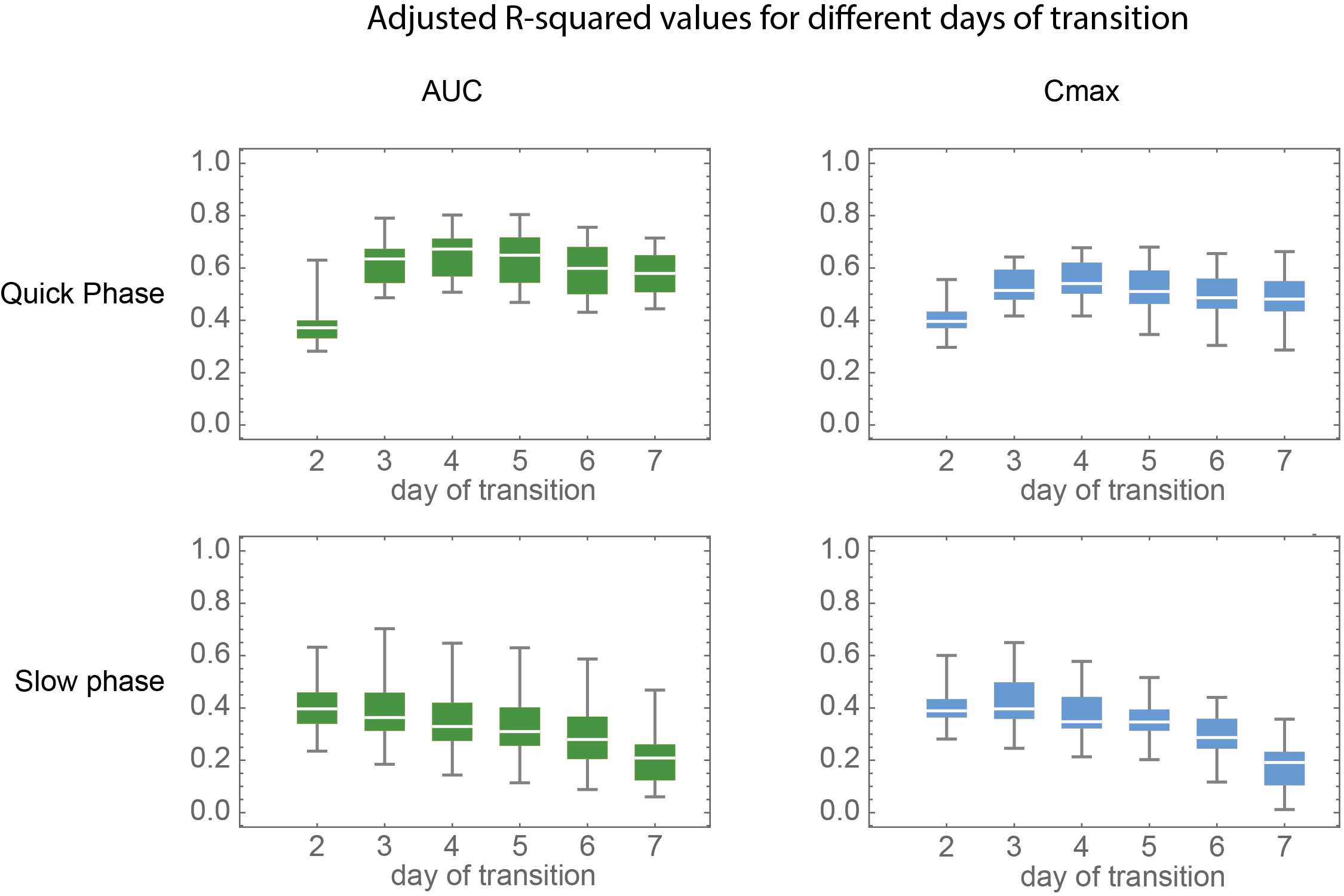

Supplement: S2 Fig — The different box-whisker plots in each figure correspond to a different set day of transition. These plots both show that values are consistently better predictors for the slope of the slow phase (based on adjusted R-squared values), as well as that we achieve the best fits for days 3 and 4. (TIF) [file pcbi.1011000.s002.tif]

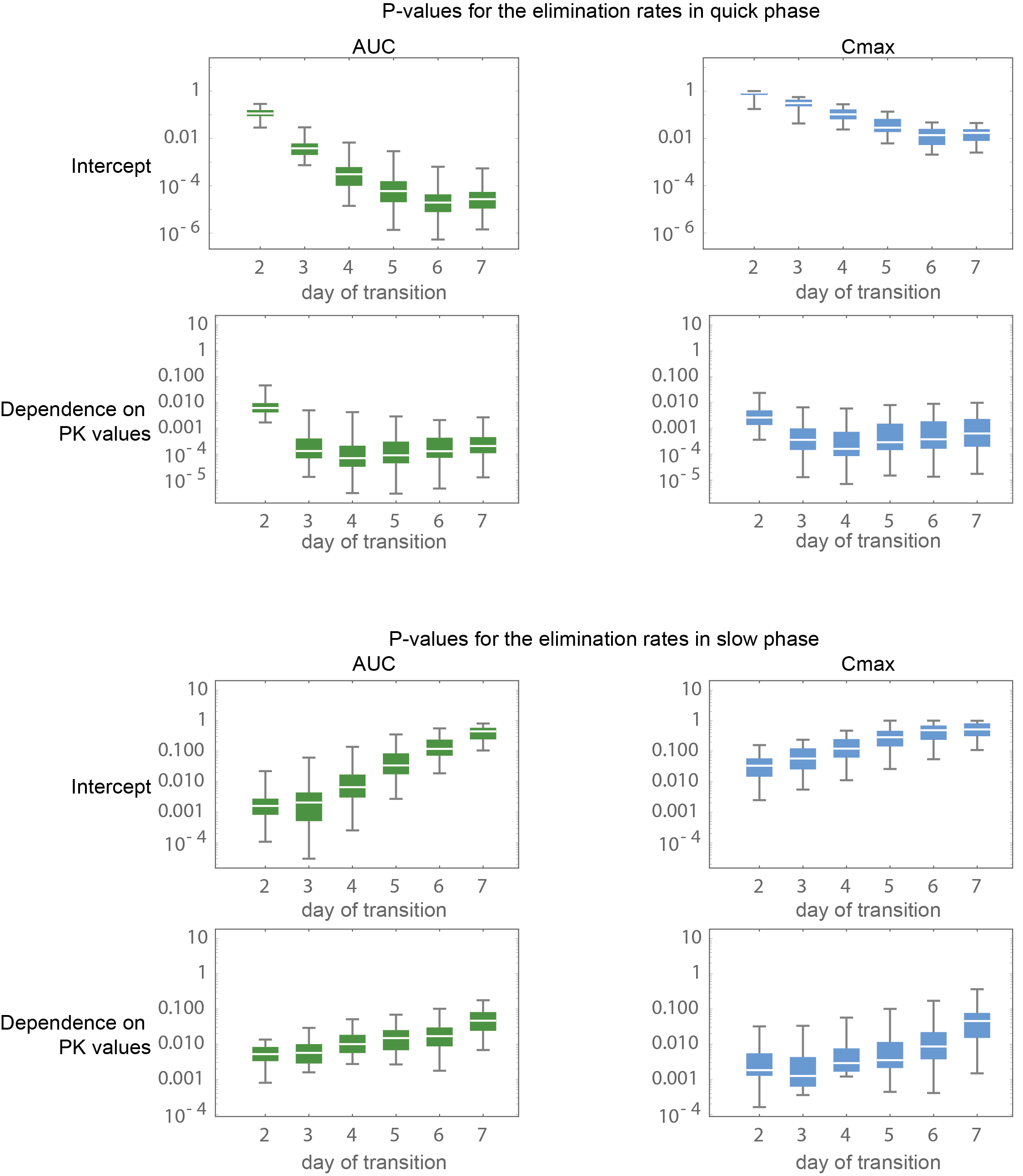

Supplement: S3 Fig — The box-whisker plots represent the results from groupings from dividing the range of PK values into 10–40 equal intervals. These plots show that if we get the best fits if the days of transition are set to day 3 or 4. Furthermore these also show that Cmax is consistently a better predictor for the slope of the slow phase than the AUC. (TIF) [file pcbi.1011000.s003.tif]

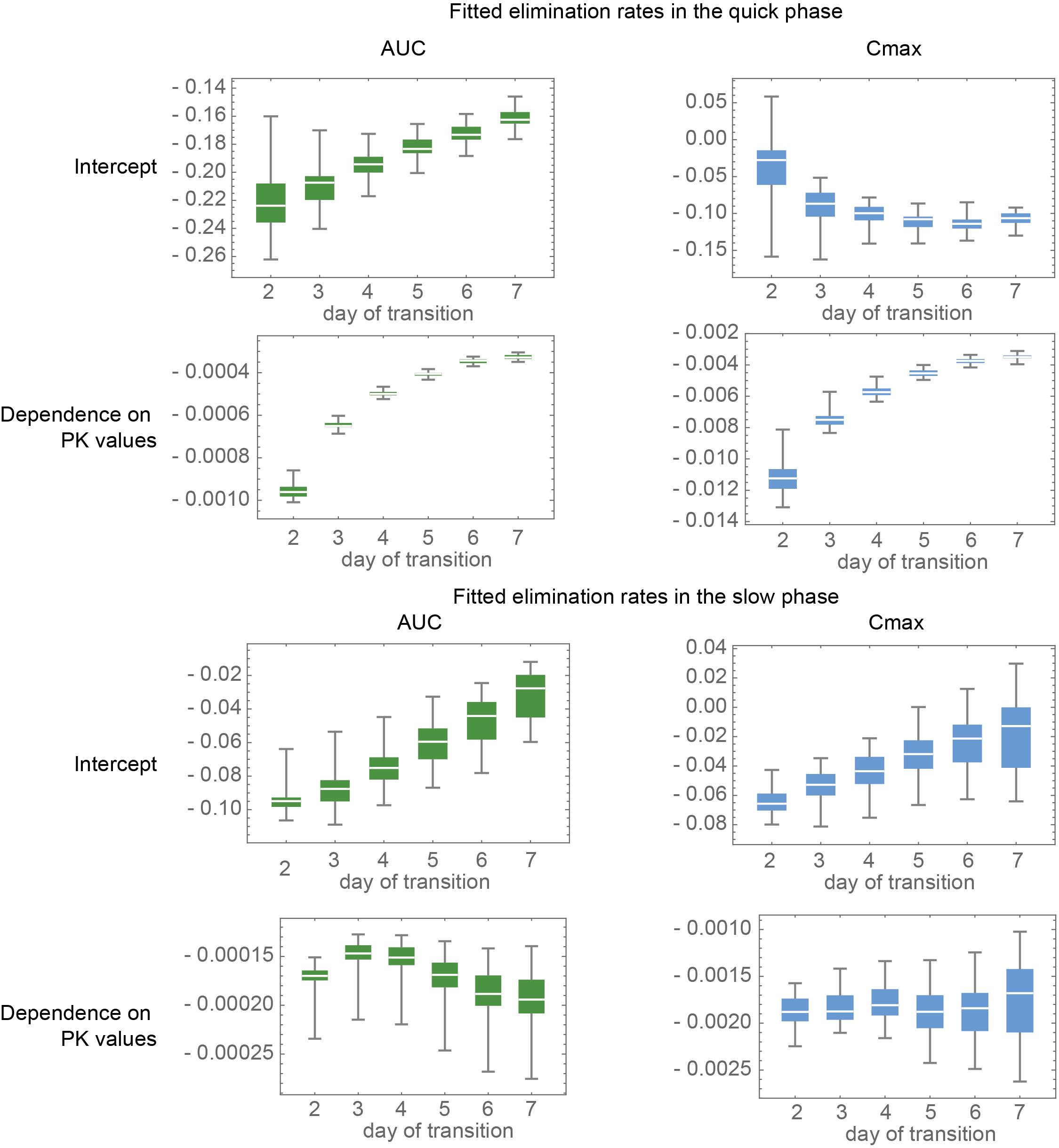

Supplement: S4 Fig — The box-whisker plots represent the results from groupings from dividing the range of PK values into 10–40 equal intervals. (TIF) [file pcbi.1011000.s004.tif]

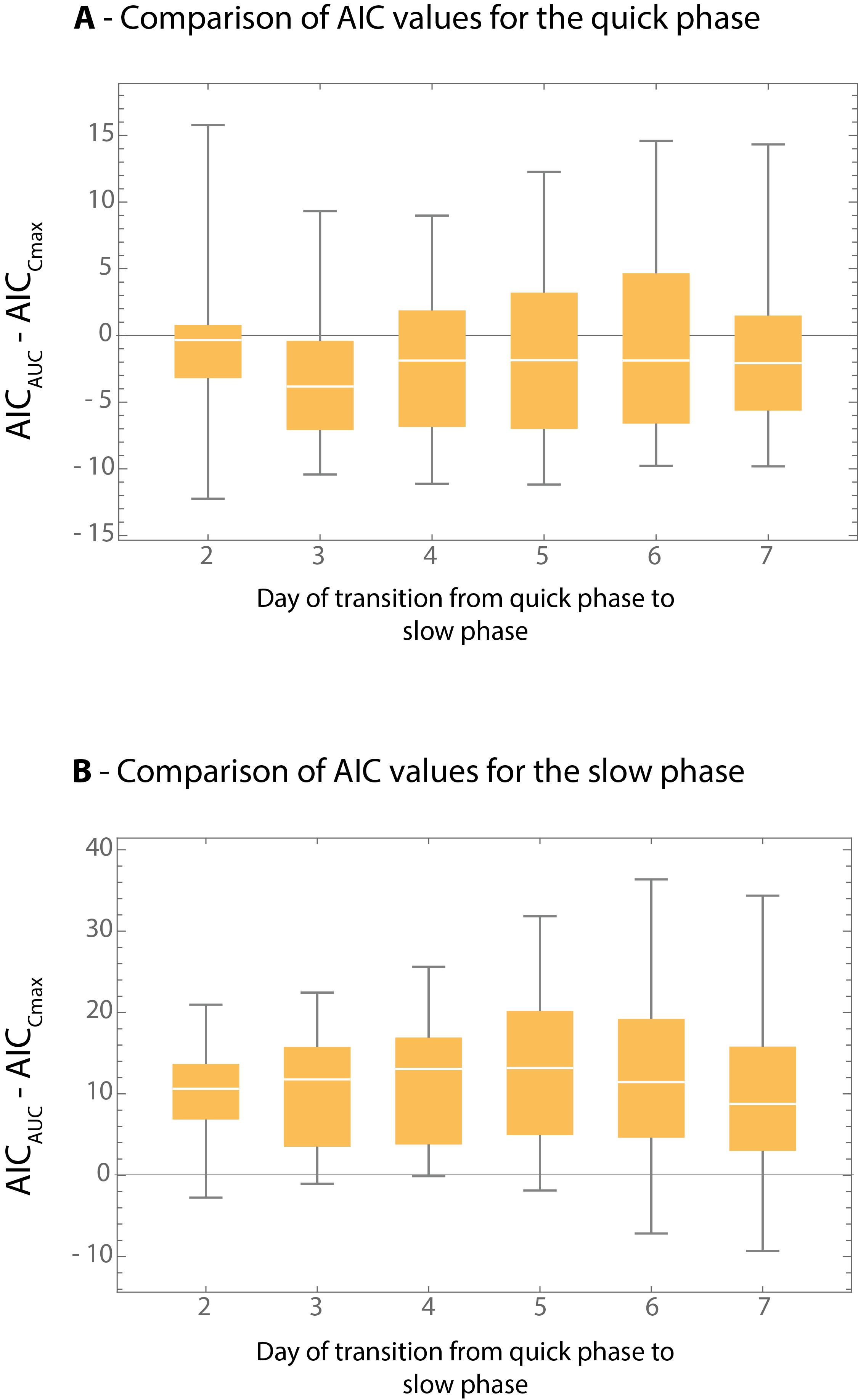

Supplement: S5 Fig — Here, the positive values indicate that Cmax is a better predictor, while negative values would indicate that AUC is a better predictor for the given phase. (TIF) [file pcbi.1011000.s005.tif]

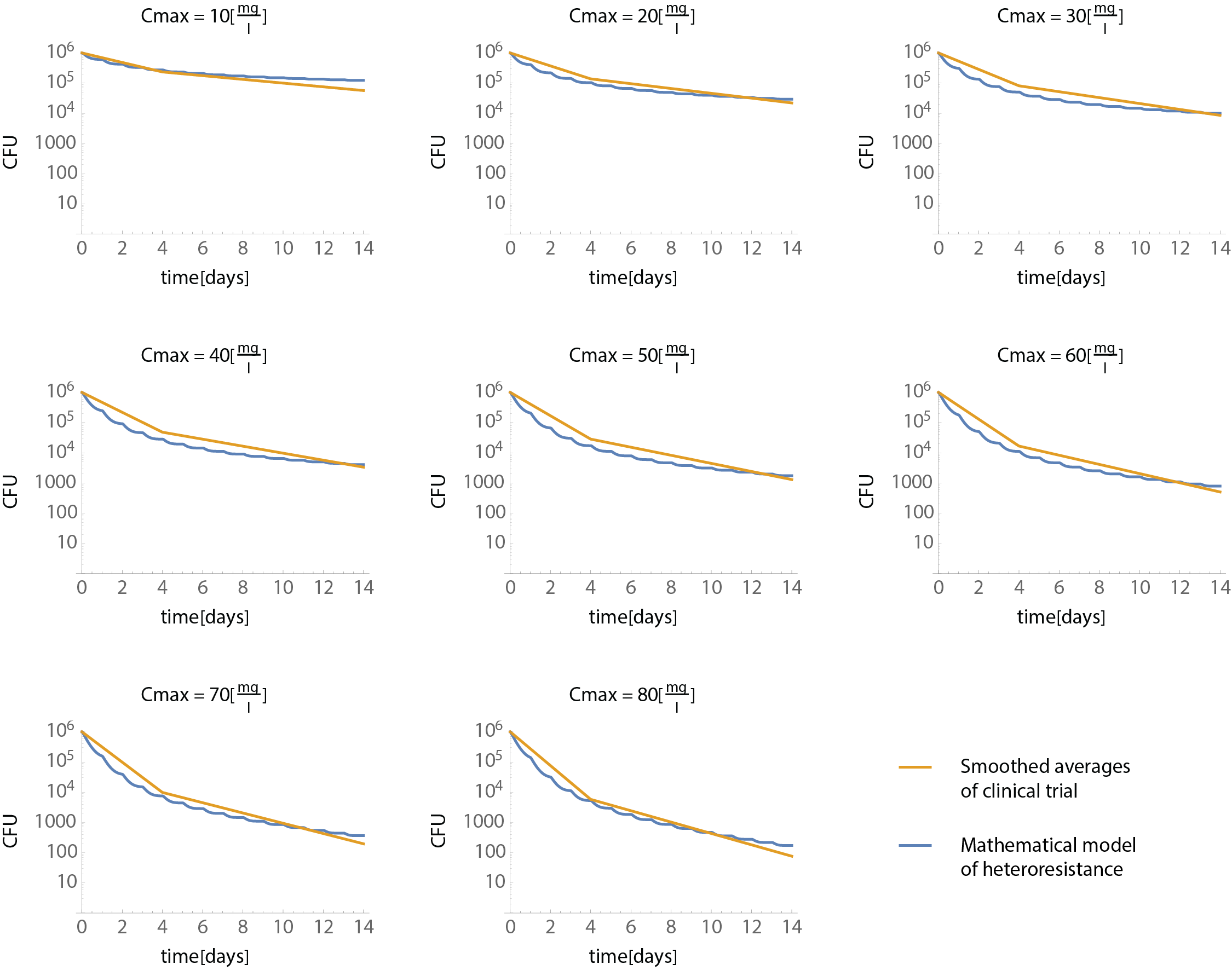

Supplement: S6 Fig — All figures show the observed/predicted bacterial counts (Y-axis) over time (X-axis). Each figure shows the same at different Cmaxes. The Cmax-es were chosen to be at regular intervals within the range of the clinical trial dataset (10–80 mg/l), the inputs doses for the mathematical models were chosen to achieve the same Cmaxes within the model. (TIF) [file pcbi.1011000.s006.tif]

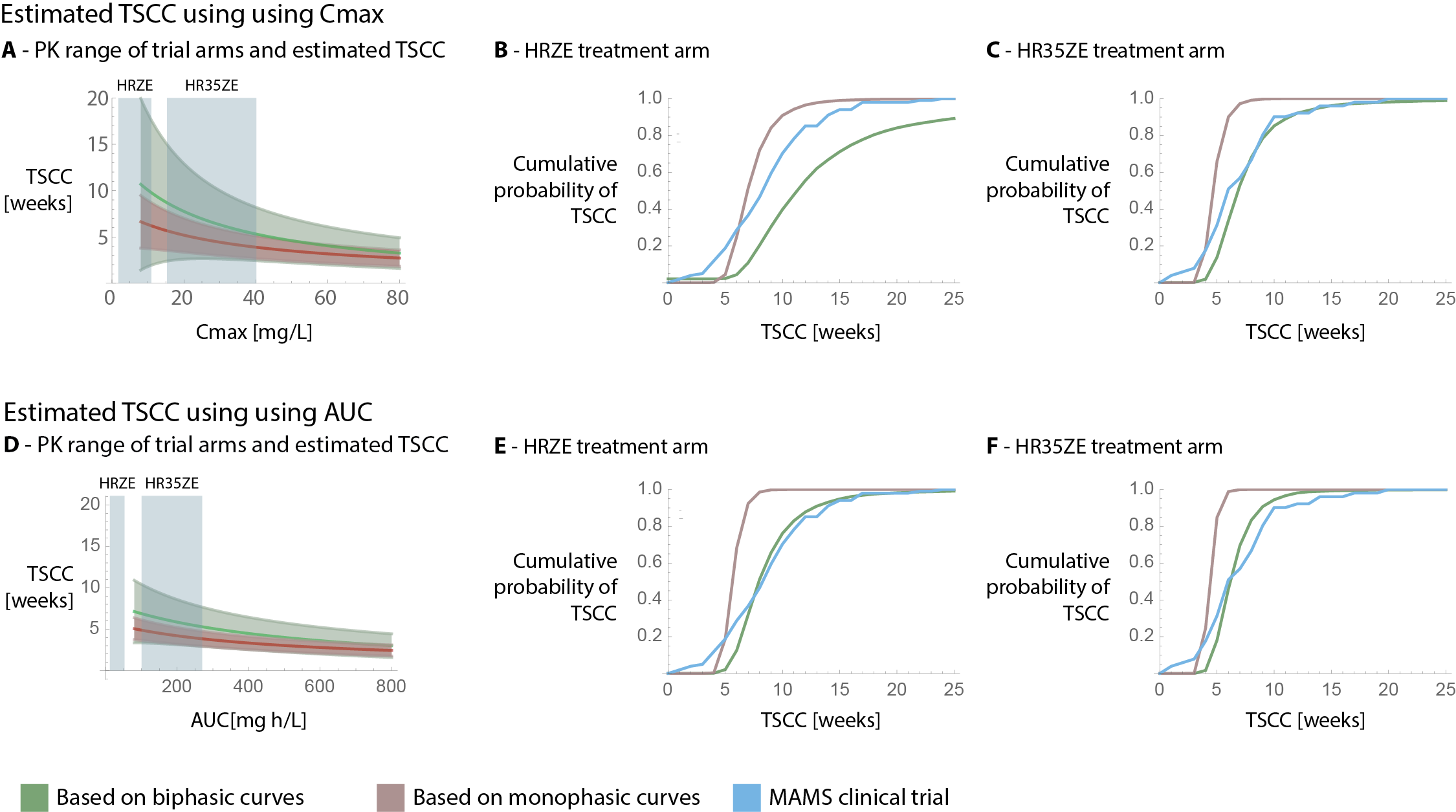

Supplement: S7 Fig — Note: this the left and right columns of this figure are the same as Fig 6. but extended with the standard dosing group for comparison. There, estimates relied on extrapolation outside the range of PK values of the EBA trials (see S7A and S7D Fig). On all of the plots the green curves show estimates based on biphasic curves (i.e taking the slowdown in decline into account), while red shows estimation based on monophasic curves (i.e. neglecting the possibility of a slowdown in decline). Blue always corresponds to data from the MAMS clinical trial. S7A and S7D Fig show the dependence of the predicted TSCC on the pharmacokinetic parameters (Cmax and AUC respectively), as well as the measured pharmacokinetic ranges for the HRZE and HR35ZE treatment arms in the MAMS clinical trial used for comparison (blue boxes). Here, the area around the estimates signify the 95% confidence interval around our estimates. The estimates are only shown within the parameter ranges of the EBA clinical trial. S7B, S7C, S7E, and S7FF Fig show the cumulative probability (Y-axis) of TSCC (X-axis) based on the estimates within the PK ranges of the MAMS trial as well as the data from the MAMS trial itself. This is shown for both Cmax and AUC, as well as the HRZE, and HR35ZE treatment arms. (TIF) [file pcbi.1011000.s007.tif]
