# Supplementary material for: High rifampicin peak plasma concentrations accelerate the slow phase of bacterial decline in tuberculosis patients: Evidence for heteroresistance
Source: PLoS Comput Biol. 2023 Apr 13;19(4):e1011000. doi: 10.1371/journal.pcbi.1011000 (PMC10128972; doi:10.1371/journal.pcbi.1011000)
Supplement: S1 Table — All the P values reported are corrected for multiple testing with the Benjamini-Hochberg method. (DOCX) [file pcbi.1011000.s008.docx]

| Property | Grouped by | Intercept  (SE) | Slope  (SE) | Intercept p-value  (corrected for multiple testing) | Slope p-value (corrected for multiple testing) | R^2^  (adjusted) | AIC  (corrected) |
| --- | --- | --- | --- | --- | --- | --- | --- |
| Elimination rates (monophasic) | AUC | -0.11  $(\pm0.016)$ | -0.00026  $(\pm0.00004)$ | ${10}^{-6}$ | ${10}^{-5}$ | 0.68 | -63 |
|  | C_max_ | -0.076  $(\pm0.02)$ | -0.0026  $(\pm0.0004)$ | 0.0017 | ${10}^{-4}$ | 0.65 | -73 |
| Baseline  (monophasic)  Log(CFU)  [ - ] | AUC | 5.34  $(\pm0.21)$ | 0.001  $(\pm0.00047)$ | ${10}^{-14}$ | 0.05 | 0.15 | 30 |
|  | C_max_ | 5.1  $(\pm0.25)$ | 0.0012  $(\pm0.005)$ | ${10}^{-14}$ | 0.02 | 0.12 | 41 |
